# Supplementary material for: Seminoma and Embryonal Carcinoma Footprints Identified by Analysis of Integrated Genome-Wide Epigenetic and Expression Profiles of Germ Cell Cancer Cell Lines
Source: PLoS One. 2014 Jun 2;9(6):e98330. doi: 10.1371/journal.pone.0098330 (PMC4041891; doi:10.1371/journal.pone.0098330)
Supplement: File S1 — ZIP file containing DMRforPairs output for significant regions. Please start from the html files. (ZIP) [file pone.0098330.s008.zip › figures/11889.pdf]

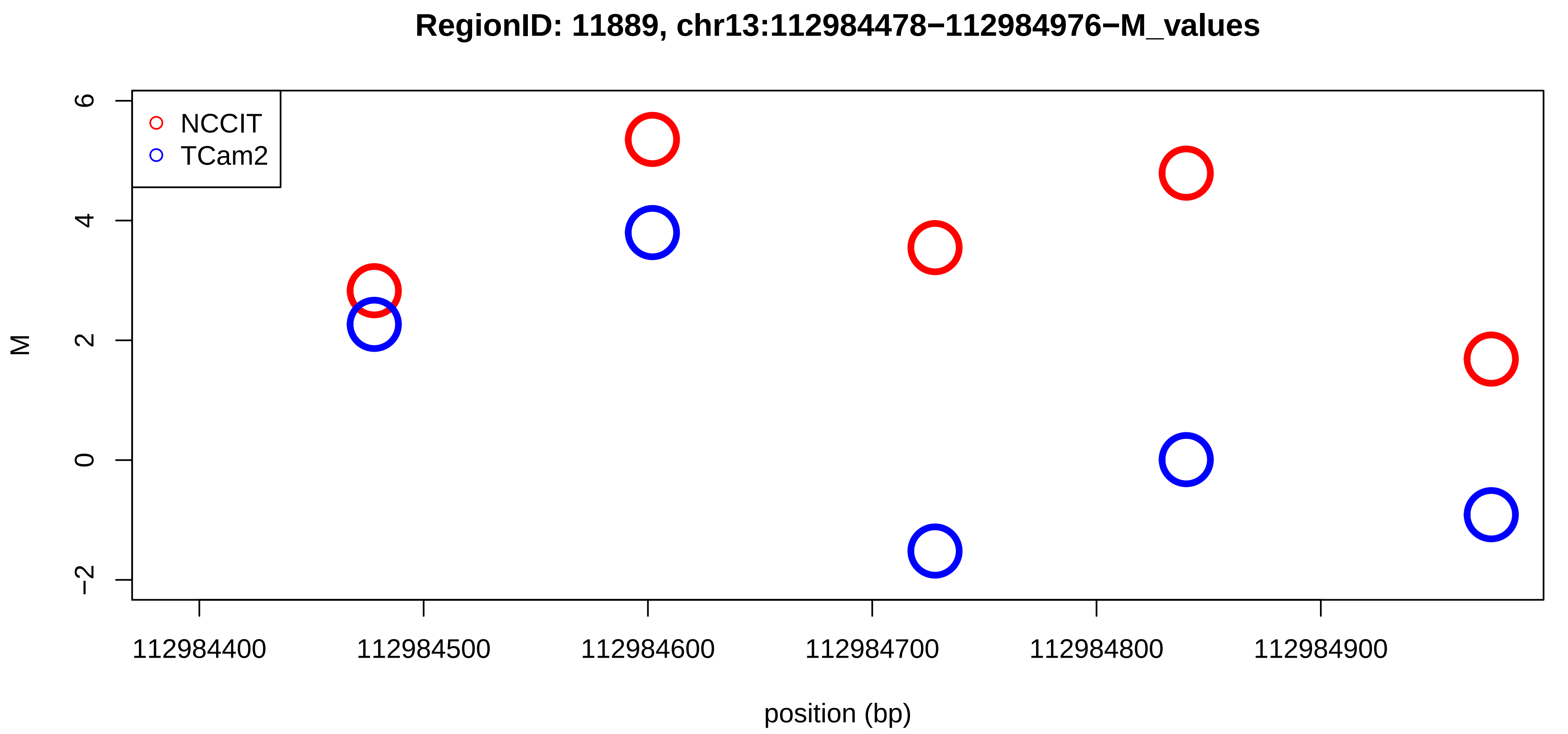
RegionID: 11889, chr13:112984478−112984976−M_values
M
−2
0
2
4
6
NCCIT
TCam2
112984400
112984500
112984600
112984700
112984800
112984900
position (bp)

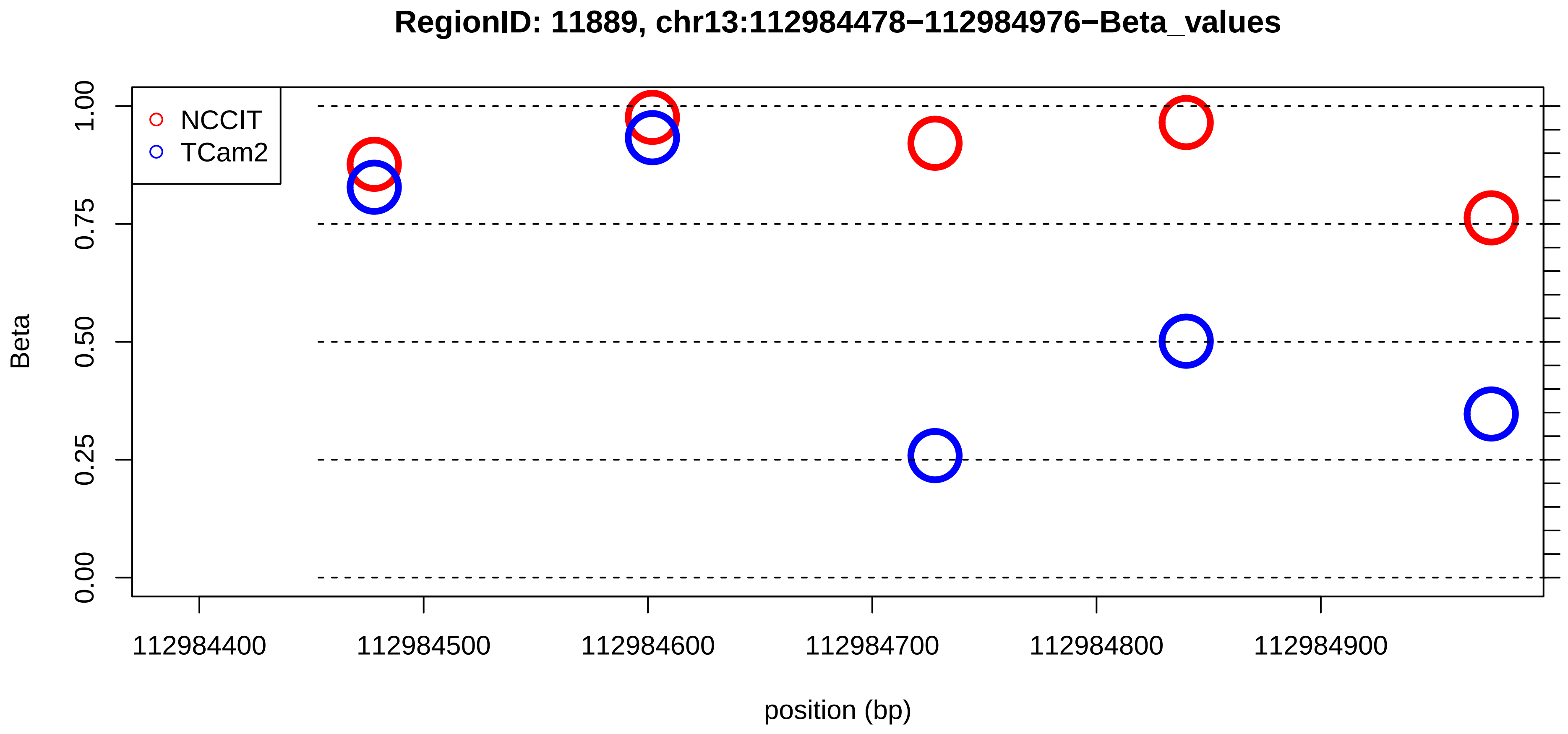
RegionID: 11889, chr13:112984478−112984976−Beta_values
Beta
0.00
0.25
0.50
0.75
1.00
NCCIT
TCam2
112984400
112984500
112984600
112984700
112984800
112984900
position (bp)
